# The Exchangeable Copper–Zinc Ratio Links Sex Hormones, Tumor Burden, and Epithelial Remodeling in Colorectal Cancer

**DOI:** 10.3390/biom16060878

**Published:** 2026-06-15

**Authors:** Rosanna Squitti, Anastasia De Luca, Altea Severino, Gianluca Rizzo, Luca Emanuele Amodio, Federica Marzi, Gabriella Vicano, Mauro Cozzolino, Angela Lombardi, Mauro Rongioletti, Vincenzo Tondolo

**Affiliations:** 1 Department of Laboratory Science, Research and Development Division, Ospedale Isola Tiberina—Gemelli Isola, 00186 Rome, Italy; altea.severino@fbf-isola.it (A.S.); maurociroantonio.rongioletti@fbf-isola.it (M.R.); 2 Department of Theoretical and Applied Sciences, eCampus University, Viale Massenzio Masia, 26, 22100 Novedrate, Italy; angela.lombardi@unicampania.it; 3 Department of Biology, University of Rome Tor Vergata, Via della Ricerca Scientifica 1, 00133 Roma, Italy; 4 Unit of Laboratory Medicine, University Hospital Tor Vergata, Via Montpellier 1, 00133 Rome, Italy; 5 UOC Chirurgia Digestiva e del Colon-Retto, Ospedale Isola Tiberina—Gemelli Isola, 00186 Rome, Italy; gianluca.rizzo@fbf-isola.it (G.R.); lucaemanueleamodio@virgilio.it (L.E.A.); federica.marzi@fbf-isola.it (F.M.); vincenzo.tondolo@fbf-isola.it (V.T.); 6 Digestive Surgery Unit, Department of Translational Medicine, Catholic University of the Sacred Heart, 00168 Rome, Italy; 7 PhD Program in Cellular and Molecular Biology, Department of Biology, University of Rome Tor Vergata, Via della Ricerca Scientifica 1, 00133 Rome, Italy; gabri.vicano@gmail.com; 8 Institute of Translational Pharmacology, CNR, Via Fosso del Cavaliere 100, 00133 Rome, Italy; mauro.cozzolino@ift.cnr.it; 9 U.P. Cytometric and Mutational Diagnostics, Vanvitelli Hospital, University of Campania “Luigi Vanvitelli”, 83031 Naples, Italy

**Keywords:** copper, zinc, colorectal cancer, sex hormones, cadherin

## Abstract

Copper (Cu)–zinc (Zn) imbalance has been implicated in colorectal cancer (CRC). Exchangeable copper (exCu), the labile circulating Cu fraction, may better reflect functionally relevant metal dysregulation than total Cu. We investigated sex-specific associations between systemic Cu–Zn indices, tumor burden, and epithelial–mesenchymal transition (EMT)-related tissue remodeling in CRC. We studied 152 CRC patients and 140 healthy controls. Serum Cu, Zn, and exCu were measured using validated analytical methods; circulating gonadotropins, sex steroids, and carcinoembryonic antigen were also assessed. EMT-related proteins (E-cadherin, vimentin, fibronectin, vinculin, MEMO1) were quantified by Western blot in paired tumor and adjacent mucosa. Analyses were sex-stratified and age-adjusted. CRC patients exhibited higher serum Cu and exCu and lower Zn than controls, resulting in a marked increase in the exCu:Zn ratio in both sexes. In patients, exCu:Zn was associated with tumor burden and pathological stage, with stronger associations with tumor size and pT stage in women and with metastatic status in men. Serum exCu:Zn was associated with tumor − normal differences in EMT-related proteins, particularly ΔE-cadherin, in both sexes. Systemic Cu–Zn disequilibrium, summarized by the exCu:Zn ratio, was associated with tumor burden and epithelial remodeling in CRC in a sex-specific manner, suggesting its potential as a biologically informative biomarker warranting further validation.

## 1. Introduction

Colorectal cancer (CRC) remains a major cause of cancer morbidity and mortality worldwide. Beyond genetic alterations, CRC is characterized by disturbances in metabolic and redox homeostasis, including dysregulation of the essential transition metal copper (Cu) and zinc (Zn) [1,2]. Both metals serve as cofactors for antioxidant enzymes, and Zn functions as Zn-finger transcription factors. They also contribute to the structural integrity of adhesion and cytoskeletal proteins relevant to epithelial plasticity [3,4,5].

In the circulation, Cu is partitioned between a tightly protein-bound fraction and a more labile, exchangeable pool (exchangeable copper, exCu). ExCu is loosely coordinated to albumin, low-molecular-weight ligands, and peripheral sites on ceruloplasmin and can actively participate in redox reactions and metal–protein exchange [1]. Zn, in turn, competes with Cu for binding to metallothioneins and Zn-dependent proteins; Zn deficiency and altered Zn metabolism have been repeatedly documented in cancer and, specifically, in CRC [5,6]. Ratios integrating labile Cu and total serum Zn, such as the exchangeable copper-to-zinc ratio (exCu:Zn), may therefore capture functionally relevant shifts in metal homeostasis more accurately than total Cu or Zn alone.

In a previous pilot study, we showed that exCu and exCu:Zn were strongly altered in CRC and that exCu:Zn discriminated patients from healthy controls with high diagnostic accuracy [7]. However, it remains unclear whether this metal disequilibrium is merely a correlate of disease status or is biochemically linked to processes implicated in tumor progression, such as epithelial–mesenchymal transition (EMT) [8,9,10]. EMT and related hybrid epithelial states involve coordinated remodeling of adhesion and cytoskeletal complexes, including E-cadherin, vimentin, fibronectin, and vinculin [11,12]. These proteins are sensitive to redox conditions and metal-dependent signaling, and their organization can be further modulated by sex hormones and gonadotropins [13].

Given the known interplay between sex hormones and systemic Cu–Zn homeostasis (e.g., estrogen-related modulation of circulating Cu) and reported sex differences in Cu/Zn carriers, we measured gonadotropins and sex steroids to explore whether endocrine background might contribute to sex-stratified patterns observed for exCu:Zn [14].

In this study, we quantified Cu, Zn, exCu, and derived indices, together with sex hormones, in serum and EMT-related proteins in paired tumor and adjacent mucosa, in a well-characterized CRC cohort with sex-stratified analyses. We hypothesized that Cu-Zn disequilibrium, summarized by exCu:Zn, is associated with clinical and tissue-level features of disease in a sex-stratified pattern. Our primary objective was to test whether exCu:Zn is associated with CRC status and clinical measures of tumor burden and pathological stage in sex-stratified analyses. As secondary, tissue-based analyses, we assessed whether this systemic signature paralleled local epithelial remodeling by quantifying EMT-related proteins in paired tumor and matched adjacent mucosa (Δ = tumor − adjacent). Finally, as exploratory analyses, we examined associations with circulating gonadotropins/sex steroids (and CEA as a clinical tumor marker) to contextualize sex-specific patterns.

## 2. Materials and Methods

### 2.1. Subjects

Patients with colorectal cancer (CRC) were consecutively enrolled at the Digestive and Colorectal Surgery Unit of the Isola Tiberina Hospital—Gemelli Isola between October 2023 and January 2025. Eligibility criteria included age > 18 years and histologically confirmed colorectal adenocarcinoma, in accordance with the 2023 NCCN Guidelines for Colon Cancer. All patients underwent standard staging procedures at diagnosis.

Healthy controls consisted of 140 volunteer blood donors recruited at the Ematos–FIDAS Unit of the same institution between October 2023 and May 2025, following standard donor screening and exclusion of major chronic comorbidities. They were not individually matched to CRC patients for age or sex; therefore, analyses were performed in a sex-stratified manner and adjusted for age when appropriate. This reflects the real-world availability of control samples suitable for biochemical analyses. In addition, a sensitivity analysis was performed by restricting the control group to individuals aged ≥60 years to improve comparability with the CRC cohort. The results of this analysis are reported in Section 3 and Supplementary Materials. For both CRC patients and controls, individuals with relevant systemic diseases or recent alcohol abuse were excluded.

Peripheral venous blood was collected at enrolment from all participants to obtain serum for measurement of sex hormones (FSH, LH, 17β-estradiol, progesterone, testosterone), carcinoembryonic antigen (CEA), and Cu–Zn–related indices (total Cu, Zn, and exCu). In CRC patients, paired samples of primary tumor tissue and matched adjacent non-neoplastic mucosa were obtained at surgery and used for EMT-related protein analysis by Western blot and for tissue copper determination by GF-AAS. Approximately 40% of participants had hormone measurements previously reported in a study on iron–hormone interactions in CRC [13].

The study was conducted in accordance with the Declaration of Helsinki and approved by the Institutional Ethics Committee (CET Lazio, Area 3) of the Fondazione Policlinico Universitario A. Gemelli IRCCS—Università Cattolica del Sacro Cuore (protocol No. 6091/2023; approval date 26 October 2023). All participants provided written informed consent.

### 2.2. Colorectal Cancer Staging

Tumor staging was based on the TNM system, with the primary tumor (T), regional lymph nodes (N), and distant metastases (M) evaluated during the histopathological examination of the surgical specimens obtained at CRC surgery [15,16,17]. Histopathological assessment was performed in accordance with the guidelines of the College of American Pathologists (CAP) (2014; https://www.cap.org/protocols-and-guidelines/cap-guidelines, accessed on 1 May 2026) and the Royal College of Pathologists (2014; https://www.rcpath.org/profession/guidelines.html, accessed on 1 May 2026). The TNM categories were assigned following standardized criteria for colorectal cancer, considering the depth of tumor invasion, the extent of nodal involvement, and the presence or absence of distant metastases, as summarized in contemporary TNM classification references [15,16,17].

### 2.3. Analytical Methods

Serum Cu- and Zn-related biomarkers were measured using validated protocols previously described [7,18]. Total serum Cu and Zn were quantified by commercial colorimetric assays (Randox; Sentinel CH Spa, Milan, Italy), with approximately 30% of samples re-analysed by graphite furnace atomic absorption spectrometry (GF-AAS; THGA AAnalyst 600, PerkinElmer, Waltham, MA, USA) to verify analytical accuracy. Agreement between methods was high, with Bland–Altman bias values < 3.6% for both metals. Exchangeable copper (exCu) was quantified by ultrafiltration followed by GF-AAS of the ultrafiltrate, as previously described [7,18]. Certified reference sera (Seronorm™ Trace Elements Serum L-1 and L-2) were used as external quality controls.

Serum gonadotropins and sex hormones (FSH, LH, estradiol, progesterone, testosterone), as well as carcinoembryonic antigen (CEA), were measured using chemiluminescent microparticle immunoassays (CMIA). Assay calibration and performance were verified using pooled reference control sera (Multichem S Plus, Technopath Clinical Diagnostics, Ballina, Co. Tipperary, V94 FF1P, Irlanda).

### 2.4. Tissue Homogenization Western Blot Analysis of Tissue Homogenates

Tumor and matched adjacent non-neoplastic mucosal samples were homogenized using a stainless-steel bead-based system (Qiagen Tissuelyser II, Qiagen, Milan, Italy) in lysis buffer, according to the manufacturer’s instructions. Homogenates were clarified by centrifugation, and supernatants were collected for protein analysis; parallel aliquots were digested in 60% HNO_3_ for subsequent metal quantification by GF-AAS. Total protein content was measured using a Lowry-based colorimetric assay (DC™ Protein Assay Kit, Bio-Rad, Hercules, CA, USA).

Equal amounts of protein (50 µg) were mixed with 4× Laemmli buffer (50 mM Tris–HCl pH 7.4, 1 mM EDTA, 1 mM EGTA, 1% Triton X-100, 10 mM NaF, 1 mM Na_3_VO_4_, protease inhibitors and 5% β-mercaptoethanol), heated at 95 °C for 5 min, and separated by SDS–PAGE on 8–12% polyacrylamide gels according to target protein molecular weight. Proteins were transferred onto PVDF membranes (Bio-Rad, Hercules, CA, USA) under standard conditions. Primary antibodies, their sources, and working dilutions have been previously described [19]. Membranes were incubated with HRP-conjugated secondary antibodies (Sigma-Aldrich, Saint Louis, MO, USA), and immunoreactive bands were detected using enhanced chemiluminescence (Clarity Max™, Bio-Rad, Hercules, CA, USA) and acquired with a ChemiDoc MP Imaging System, following procedures previously applied to CRC tissues [19]. Antibody specificity had been validated in human colorectal, pancreatic, and breast cancer cell lines [19]. Band intensities were quantified by densitometry using ImageJ software (version 1.54M), with β-actin used as the internal loading control.

### 2.5. Statistical Analyses

Serum hormone concentrations (FSH, LH, estradiol, progesterone, testosterone), CEA, and Cu–Zn–related indices (total Cu, Zn, Cu:Zn ratio, exCu, exCu:Zn ratio, and tissue Cu) were examined for distributional properties and, due to skewed distributions, are reported as median and interquartile range (IQR). Unadjusted differences between CRC patients and healthy controls were assessed using non-parametric tests (Mann–Whitney U). For case–control comparisons, age-adjusted *p*-values were obtained from sex-stratified linear regression models including study group (CRC vs. control) as the independent variable and age as a covariate. Sensitivity analyses additionally included hemoglobin as a covariate for total Cu, Zn, and the Cu:Zn ratio. For EMT-related markers measured in paired tumor and adjacent mucosa, within-subject differences were evaluated using the Wilcoxon signed-rank test, stratified by sex. A two-sided *p* < 0.05 was considered statistically significant.

Sex-stratified correlations were assessed using Spearman’s rank test [Spearman’s ρ (rho)]. Correlation strength was interpreted as follows: ρ values < 0.30 were considered weak, values between 0.30 and 0.49 moderate, and values ≥ 0.50 strong correlations. For analyses including both CRC patients and controls, age effects were accounted for by computing correlations on age-residualized values; hemoglobin was additionally included when appropriate for total Cu, Zn, and Cu:Zn ratio. For analyses restricted to CRC patients, correlations were computed on raw or hemoglobin-residualized values as specified in the Results. Correlation analyses examined associations between: (i) Cu–Zn indices, circulating hormones, and CEA; (ii) Cu–Zn indices and EMT-related markers using tumor − normal differences (Δ = tumor − adjacent mucosa); and (iii) Cu–Zn indices and clinical tumor parameters (tumor size, pathological stage, metastasis, and TNM stage). Correlation matrices were visualized as sex-specific heatmaps displaying Spearman’s ρ (rho) values, with significant correlations (*p* < 0.05) indicated. Given the strong intercorrelations among Cu–Zn indices and the exploratory, hypothesis-driven nature of the analyses, no formal correction for multiple testing was applied, and results should be interpreted as exploratory; therefore, all reported *p*-values should be interpreted as nominal significance only. Statistical analyses were performed using SPSS (version 16.0) and GraphPad Prism (version 9.3.1).

### 2.6. Study Outcomes and Analytical Strategy

The primary outcome was the association of systemic Cu–Zn disequilibrium (exCu:Zn) with CRC status and clinical tumor burden/stage in sex-stratified analyses. Secondary tissue-based outcomes included paired tumor − adjacent differences in EMT-related proteins and tissue Cu (Δ = tumor − adjacent mucosa) and their associations with systemic Cu–Zn indices. Exploratory outcomes included associations between Cu–Zn indices and circulating gonadotropins/steroid hormones; CEA was analyzed as a clinical tumor marker. Statistical details for each outcome are reported below.

## 3. Results

Results are presented from systemic case–control comparisons to clinical correlates of tumor burden, to tissue-level paired differentials, and finally, to exploratory endocrine associations.

### 3.1. Study Population and Baseline Characteristics

A total of 292 participants were included in the study, comprising 152 patients with CRC and 140 healthy controls.

The clinicopathological characteristics of the CRC cohort are summarized in Table 1. The majority of tumors were classified as pT3, followed by pT2 and pT4 stages, with a smaller proportion of early stage tumors (pT1). Most patients were node-negative (pN0), although a substantial proportion showed nodal involvement (pN1–pN2). Distant metastases were present in a minority of cases. Tumor location included both right- and left-sided colon cancers, with representation of both right- and left-sided tumors, as well as rectal cancers, reflecting the heterogeneity of the cohort. The distribution of tumor location was comparable between sexes, with no clear predominance of right-sided tumors in female patients compared to males.

The CRC group had a significantly higher mean age compared with controls (71.7 ± 12.0 vs. 56.0 ± 14.3 years, *p* < 0.001, unpaired *t*-test; Table 1). The proportion of female participants was similar in the two groups (45% in the CRC group vs. 41% in controls), and this difference was not statistically significant (*p* = 0.69, chi-square test). BMI did not differ significantly (25.7 ± 5.41 vs. 25.12 ± 6.0; *p* = 0.39). Therefore, all statistical models included age as a covariate. Since anemia is a common feature of CRC [20,21], hemoglobin levels were assessed (Table 1). To minimize the potential confounding effect of anemia, hemoglobin was additionally included as a covariate in sensitivity analyses for total serum Cu, Zn, and the Cu:Zn ratio, given the moderate associations observed between these indices and hemoglobin. We then compared systemic Cu–Zn indices and circulating markers between CRC patients and controls in sex-stratified analyses.

### 3.2. Systemic Cu–Zn Indices and Endocrine Markers in CRC: Case–Control Comparisons

We first compared circulating hormone levels between CRC patients and healthy controls, stratified by sex and adjusting for age (Supplementary Table S1). In females, CRC patients showed significantly lower gonadotropin levels and higher sex steroid levels compared with controls: serum FSH and LH were reduced in CRC (FSH: *p* = 0.005; LH: *p* = 0.037), whereas estradiol and progesterone were increased (*p* = 0.023 and *p* = 0.001, respectively). In contrast, the serum tumor marker CEA and testosterone did not differ significantly between female CRC patients and controls (both *p* > 0.10). In males, none of the measured hormones showed a statistically significant difference between CRC patients and controls after age-adjustment (all *p* ≥ 0.09), indicating broadly comparable hormone profiles between groups. In line with our sensitivity analyses on Cu–Zn indices, additional adjustment for hemoglobin in models including total Cu, Zn (in males), and the Cu:Zn ratio did not materially alter the pattern or significance of the main associations, suggesting that anemia has a limited impact on the relationships observed in this cohort).

We next compared Cu- and Zn-related indices between CRC patients and healthy controls, stratified by sex and adjusted for age (Table 2). In females, CRC patients showed higher total serum Cu and lower Zn levels than controls, resulting in a significantly elevated Cu:Zn ratio (all *p* ≤ 0.01) and a markedly increased exCu:Zn ratio compared with controls (*p* = 0.0011), whereas differences in exCu alone did not reach statistical significance after age adjustment. In males, differences between CRC patients and controls were even more pronounced, indicating a stronger redox-active Cu imbalance in the male cohort and a wider shift in systemic metal-binding equilibrium: total Cu and exCu were higher, Zn concentrations were substantially lower, and both the Cu:Zn and exCu:Zn ratios were strongly increased in CRC (all *p* < 0.0001), a pattern consistent with an expansion of the labile Cu pool and reduced Zn availability. To further assess the potential impact of age differences between CRC patients and controls, we performed a sensitivity analysis by restricting the control group to individuals aged ≥60 years (*n* = 62), resulting in improved age comparability with the CRC cohort (mean age 68.5 vs. 71.7 years). In this age-restricted analysis, the main findings remained unchanged. In particular, the serum exCu:Zn ratio was still markedly higher in CRC patients compared with controls aged ≥60 years (median 0.11 [IQR 0.07–0.14] vs. 0.05 [0.037–0.07]), and this difference remained highly statistically significant (Mann–Whitney U test, *p* = 1.47 × 10^−5^). Overall, these findings indicate a systemic Cu–Zn imbalance in CRC patients, consistent with disruption of thiol-rich metal homeostatic pathways and preferential loading of Cu relative to Zn. In sensitivity analyses that included hemoglobin as an additional covariate in age-adjusted models, the marked differences between male CRC patients and controls remained highly significant and in the same direction, primarily among men.

Next, within CRC patients, we examined whether Cu–Zn indices—particularly exCu:Zn—were associated with clinical measures of tumor burden and pathological stage.

### 3.3. exCu:Zn as a Systemic Correlate of Tumor Burden and Stage

In CRC patients, tumor clinical indices showed distinct sex-specific associations with Cu- and Zn-related variables (Supplementary Figure S1). In females, larger tumor size was positively correlated with total serum Cu (ρ = 0.348, *p* = 0.008) and exCu (ρ = 0.352, *p* = 0.030), consistent with an association between larger tumor size and higher circulating exCu levels. Notably, the larger the tumor size, the higher the exCu:Zn ratio (ρ = 0.454, *p* = 0.004). Conversely, a larger tumor size was inversely correlated with the tumor − normal difference in tissue Cu content (ΔCu, ρ = −0.401, *p* = 0.028).

A more advanced pathological T stage was likewise associated with higher total serum Cu (ρ = 0.355, *p* = 0.007 indicating an association between pathological T stage and circulating Cu-related indices. An advanced pathological T stage was also weakly associated with a higher Cu:Zn ratio (ρ = 0.286, *p* = 0.031), and moderately associated with increased exCu (ρ = 0.332, *p* = 0.039) and exCu:Zn ratio (ρ = 0.335, *p* = 0.037). In addition, exCu correlated positively with overall pathological TNM stage (ρ = 0.36, *p* = 0.025). No significant correlations were observed between Cu-Zn indices and pN stage or number of metastatic lymph nodes in females (all *p* > 0.05).

In males, larger tumor size was weakly associated with lower serum Zn (ρ = −0.234, *p* = 0.015) and a higher Cu:Zn ratio (ρ = 0.293, *p* = 0.011), consistent with progressive loss of Zn-binding capacity in advanced disease. A more advanced pathological T stage had a trend of correlation with serum Zn (ρ = −0.270, *p* = 0.021) and was positively associated with the Cu:Zn ratio (ρ = 0.354, *p* = 0.002). Moreover, the presence or grade of distant metastases correlated positively with exCu (ρ = 0.346, *p* = 0.016) and the exCu:Zn ratio (ρ = 0.347, *p* = 0.016) in males. No other significant correlations between Cu-Zn indices and clinical staging parameters were detected in men (all *p* > 0.05). Additional adjustment for hemoglobin in models including total Cu and the Cu:Zn ratio (and Zn in males) did not materially change the pattern or significance of the main associations .

**Figure 1:**
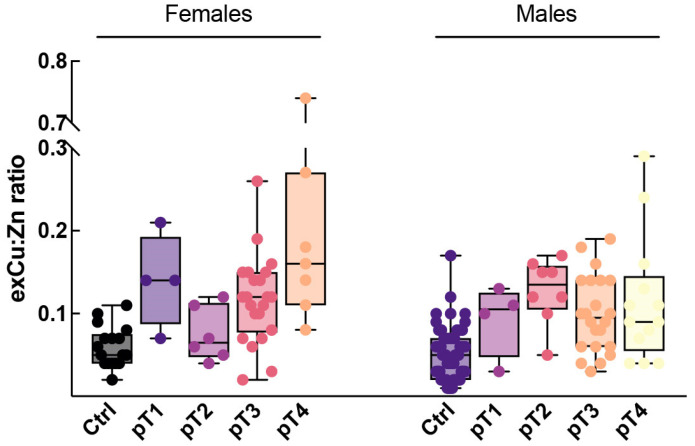
Sex-stratified distribution of serum exCu:Zn ratio according to pathological T stage in colorectal cancer (CRC) patients and healthy controls. Data are presented as box plots with individual data points overlaid. Boxes represent the interquartile range (IQR), center lines indicate medians, and whiskers indicate the data range. Analyses were stratified by sex. Sample sizes for each subgroup are indicated on the *x*-axis. An axis break was introduced in the female panel to improve visualization of subgroup distributions.

We then explored how the serum exCu:Zn ratio varied across pathological T stages, including healthy controls, stratified by sex (Figure 1). In females, median exCu:Zn values were already higher in CRC patients than in healthy controls and increased progressively with advancing pT, reaching the highest levels in pT4 tumors. This visual trend was consistent with the positive correlation between exCu:Zn and pT stage described above. In males, exCu:Zn ratios were also higher in CRC patients compared with controls, but the distribution across pT stages was more homogeneous and did not show a clear monotonic increase with advancing T stage (Figure 1).

Finally, given the sex-stratified patterns observed above, we explored associations between systemic Cu–Zn indices and circulating gonadotropins/steroid hormones, and we examined CEA as a clinical tumor marker. CEA was included as a routinely used clinical tumor marker to provide clinical context for tumor burden; analyses involving CEA were considered exploratory.

### 3.4. Sex-Specific Endocrine Background and Systemic Cu–Zn Indices (Exploratory)

In the combined sample of CRC patients and healthy controls, we next examined the age-adjusted relationships between Cu-Zn indices and circulating hormones, stratified by sex (Supplementary Figure S2). Age effects were removed by linear regression, and Spearman correlations were computed on the residuals.

In females, higher CEA levels showed a weak-to-moderate positive association with several Cu-related indices, including age-adjusted total Cu (ρ = 0.30, *p* = 0.017), the Cu:Zn ratio (ρ = 0.32, *p* = 0.014) and exCu (ρ = 0.36, *p* = 0.014). By contrast, FSH showed weak inverse associations with the Cu:Zn ratio (ρ = −0.28, *p* = 0.033) and was strongly negatively correlated with the exCu:Zn ratio (ρ = −0.54, *p* = 0.0003). In males, the only significant associations were observed with LH, which correlated negatively with Zn (ρ = −0.32, *p* = 0.004) and positively with the Cu:Zn ratio (ρ = 0.33, *p* = 0.003). There was a trend between exCu:Zn and CEA (ρ = 0.28, *p* = 0.083, and exCu:Zn and LH (ρ = −0.31, *p* = 0.070). No other age-adjusted metal–hormone correlations reached statistical significance (all *p* > 0.05; Supplementary Figure S2). When hemoglobin was introduced as an additional covariate in sensitivity analyses for total Cu, Zn (in males), and the Cu:Zn ratio, the strength and direction of the associations with circulating hormones remained essentially unchanged.

To explore whether this systemic signature paralleled local tissue remodeling, we assessed EMT-related proteins and tissue Cu in paired tumor and matched adjacent mucosa, focusing on within-patient differentials (Δ = T − A).

### 3.5. EMT-Related Proteins in Paired Tumor and Matched Adjacent Non-Neoplastic Mucosa (Δ = T − A)

**Figure 2:**
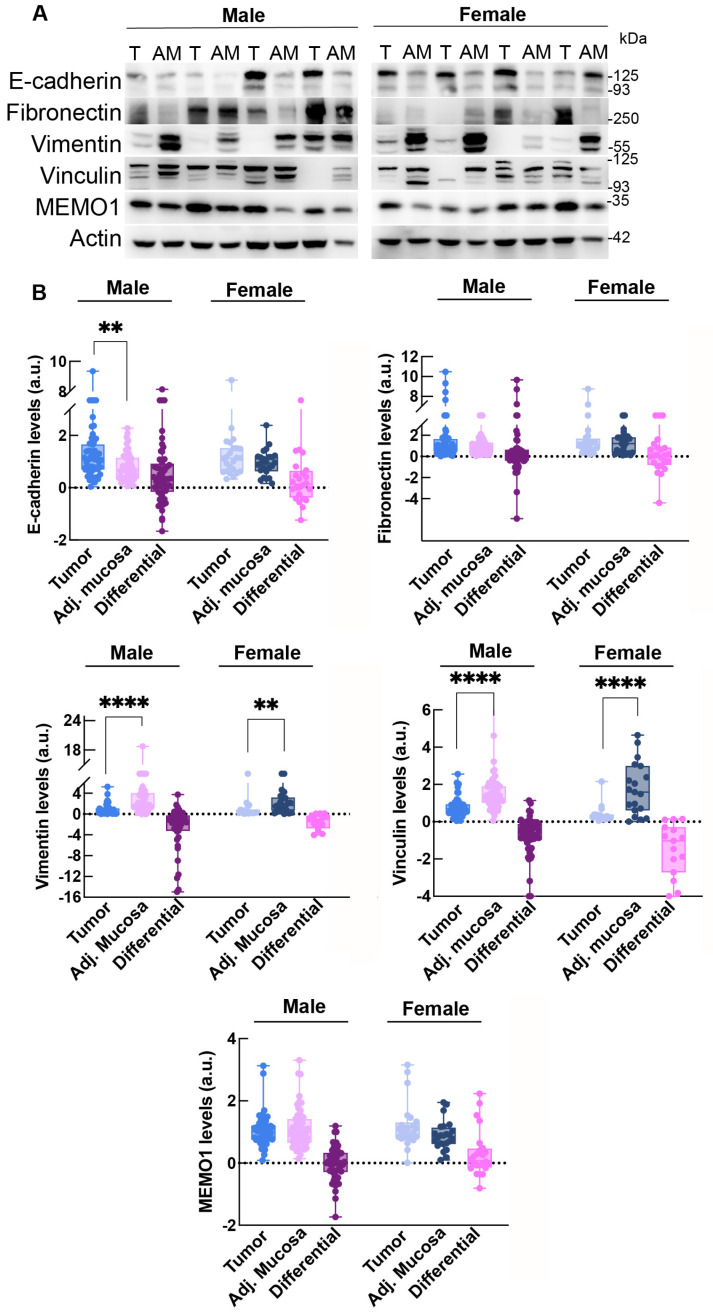
EMT-related protein expression in paired tumor (T) and matched adjacent non-neoplastic mucosa (AM) from CRC patients, and within-patient differential (Δ = Tumor − Adjacent non-neoplastic mucosa). (**A**) Representative Western blots for E-cadherin, fibronectin, vimentin, vinculin, and MEMO1 in tumor tissue (T) and matched adjacent non-neoplastic mucosa (AM), stratified by sex. β-actin was used as the loading control. (**B**) Densitometric quantification normalized to β-actin and expressed as the within-patient differential for each marker (ΔEMT = T − AM). Data are shown as box-and-whisker plots (median and interquartile range; whiskers indicate minimum and maximum). Paired comparisons were performed using the Wilcoxon signed-rank test. ** *p* < 0.01; **** *p* < 0.0001. The original western blot images can be found in Supplementary Materials.

EMT-related protein levels were compared between tumor tissue and adjacent normal mucosa in CRC patients, stratified by sex (Table 3 and Figure 2). Systemic analyses were performed on the full available cohort, whereas tissue-based analyses were conducted on the subset of patients with complete paired tissue, serum, haemoglobin, and hormone profile data. MEMO1 did not show relevant differences between tumor and normal tissue in either females or males (both *p* > 0.10). In contrast, vinculin was markedly reduced in tumor samples in both sexes: median values decreased from 1.18 (0.96–1.41) to 0.64 (0.42–1.07) in females and from 1.25 (1.02–1.89) to 0.70 (0.46–0.92) in males, with negative tumor–mucosa differences and highly significant *p*-values (*p* < 0.001 in both sexes). Fibronectin levels were broadly comparable between tumor and normal mucosa, and no significant paired differences were detected (*p* = 0.663 in females, *p* = 0.271 in males).

E-cadherin showed a sex-specific pattern: in females, tumor levels tended to be higher than in matched mucosa but did not reach statistical significance (*p* = 0.201), whereas in males, E-cadherin was significantly increased in tumor tissue (median Δ tumor–mucosa 0.33, *p* = 0.002), a finding compatible with Zn-dependent stabilization of epithelial junctional complexes despite reduced Zn availability, suggesting compensation through Cu-sensitive regulatory pathways. Vimentin, a mesenchymal marker, was consistently lower in tumor than in adjacent mucosa in both sexes, with large negative tumor–mucosa differences (Δ = −1.25 in females and −1.54 in males) and significant *p*-values (*p* = 0.006 and *p* < 0.0001, respectively). Overall, these findings show a shift toward reduced mesenchymal features (vimentin, vinculin) and increased epithelial E-cadherin expression in tumor tissue, particularly in male patients, indicating a reorganization of adhesion and cytoskeletal components whose structural stability appears to depend on redox balance and divalent-metal occupancy.

We next examined whether systemic Cu–Zn indices and tissue Cu were associated with EMT-related protein patterns, focusing on within-patient differentials (Δ = T − A) as a summary of tumor-specific remodeling.

### 3.6. Systemic Cu–Zn Indices and Tissue Remodeling: Associations with EMT-Related Proteins (Exploratory)

When EMT-related differences between tumor and adjacent normal mucosa (ΔEMT = tumor − normal) were considered, serum Cu- and Zn-related indices showed sex-specific associations. In females, ΔE-cadherin was positively correlated with total Cu (ρ = 0.42, *p* = 0.043), and inversely with Zn (ρ = −0.56, *p* = 0.005), the Cu:Zn ratio (ρ = 0.64, *p* = 0.001), exCu (ρ = 0.53, *p* = 0.015), and the exCu:Zn ratio (ρ = 0.66, *p* = 0.0017), consistent with Cu-sensitive modulation of adhesion complexes that are partially Zn-dependent for conformational stability. There was a trend of association between exCu and exCu:Zn with ΔFibronectin (ρ = 0.43, *p* = 0.057 and ρ = 0.43, *p* = 0.061, respectively). In males, ΔE-cadherin correlated selectively with exCu (ρ = 0.37, *p* = 0.036), suggesting a role for labile Cu in regulating supramolecular epithelial organization. Furthermore, ΔE-cadherin correlated with the serum exCu:Zn ratio (ρ = 0.36, *p* = 0.035), whereas higher serum Zn levels were inversely associated with ΔVimentin (ρ = −0.41, *p* = 0.006), aligning with the known role of Zn in limiting the cytoskeletal mesenchymal phenotype and preserving the epithelial phenotype (Figure 2).

**Figure 3:**
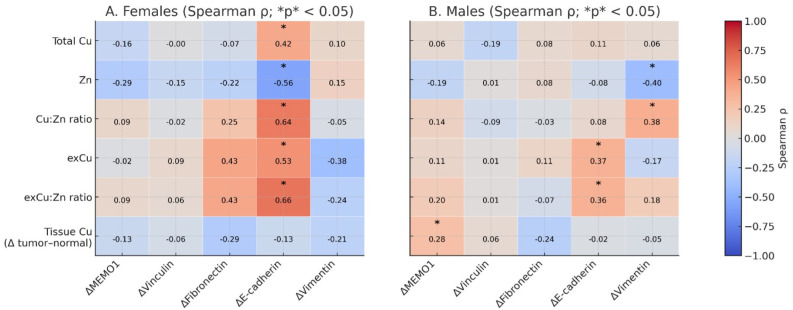
Spearman correlations between EMT-related differences (tumor − normal mucosa) and copper/zinc-related indices, stratified by sex. Heatmaps show Spearman correlation coefficients (ρ) between tumor − normal differences (Δ) in EMT-related protein expression (ΔMEMO1, ΔVinculin, ΔFibronectin, ΔE-cadherin, ΔVimentin; columns) and systemic/tissue copper–zinc indices (rows) in (**A**) females and (**B**) males with colon cancer. Copper/zinc-related variables include total serum copper (Total Cu), serum zinc (Zn), the serum Cu:Zn ratio (Cu:Zn ratio), exchangeable copper (exCu), the exchangeable copper-to-zinc ratio (exCu:Zn ratio), and the tumor − normal difference in tissue copper content (Tissue Cu (Δ tumor − normal). Colors represent Spearman ρ values (blue = negative, red = positive), as indicated by the color scale. Numerical values in each tile indicate the corresponding correlation coefficient; tiles marked with an asterisk (*) denote statistically significant correlations (*p* < 0.05). *p*-values shown in the heatmaps represent nominal significance and were not adjusted for multiple testing.

In addition, in males, tissue ΔCu showed a weak positive correlation with ΔMEMO1 (ρ = 0.28, *p* = 0.043). Further adjustment for hemoglobin in the subset of analyses involving EMT-related markers and Cu–Zn indices did not materially affect the observed correlations or their statistical significance. To further explore whether circulating Cu–Zn imbalance was independently related to EMT remodeling, we modelled the ΔE-cadherin as a function of serum exCu:Zn in CRC patients, stratified by sex (Figure 3).

In females, higher exCu:Zn was positively associated with a greater increase in E-cadherin expression in tumor tissue compared with matched mucosa (ρ = 0.66, *p* < 0.0001). A similar positive association was observed in males, although weaker in magnitude (ρ = 0.36, *p* = 0.049). Finally, given the sex-stratified patterns observed above, we explored whether circulating gonadotropins/steroid hormones were associated with EMT-related differences (Δ = T − A) as an exploratory association between endocrine background and tissue remodeling.

### 3.7. Sex-Specific Endocrine Background and ΔEMT Features in Paired Tissue (Exploratory)

For each patient, EMT marker levels and tissue Cu were quantified in matched tumor and adjacent non-neoplastic mucosa, and the primary endpoint was the within-patient differential (Δ = Tumor − Adjacent non-neoplastic mucosa). In the CRC group, we next examined the relationships between circulating sex hormones and EMT-related differences between tumor and adjacent normal mucosa (ΔEMT). In females, serum FSH levels were inversely associated with ΔFibronectin (ρ = −0.460, *p* = 0.041), indicating that higher FSH was linked to a smaller tumor − normal increase in fibronectin. No other significant correlations were observed between female sex hormones (estradiol, LH, progesterone, testosterone) and any ΔEMT marker (all *p* > 0.05). In males, serum progesterone showed a positive correlation with ΔVimentin (ρ = 0.387, *p* = 0.032), suggesting that higher progesterone levels were associated with a greater tumor − normal increase in vimentin expression. All remaining correlations between male sex hormones (FSH, estradiol, LH, testosterone) and ΔEMT markers were not statistically significant (all *p* > 0.05).

## 4. Discussion

A main finding of this exploratory study is that systemic copper–zinc disequilibrium, quantified by the exchangeable copper-to-zinc ratio (exCu:Zn), is closely associated with tumor burden and epithelial remodeling in colorectal cancer (CRC), with a clear sex-stratified pattern. Given the cross-sectional and exploratory design, these associations should not be interpreted as causal but rather as hypothesis-generating signals linking systemic metal homeostasis to clinical and tissue-level features of CRC.

In line with our previous pilot observations [19], CRC patients showed higher total serum copper and exchangeable copper, together with lower zinc levels, resulting in a marked increase in exCu:Zn compared with healthy controls. Importantly, exCu:Zn was not merely associated with disease presence but also correlated with indices of tumor burden and pathological stage. In women, higher exCu:Zn was associated with larger tumor size and more advanced pathological T stage, whereas in men it correlated with metastatic status. These findings suggest that systemic Cu–Zn imbalance is associated with severity and tumor burden. However, given the cross-sectional design, it cannot be determined whether these alterations are directly related to tumor progression or reflect broader cancer-associated metabolic and inflammatory changes.

Copper and zinc are functionally coupled through competition for thiol-rich ligands and metalloproteins, and their balance critically influences redox homeostasis and protein function [23,24]. An elevated exCu:Zn ratio integrates two complementary processes: expansion of the labile, redox-active copper pool and erosion of zinc-dependent buffering capacity. In this context, exCu:Zn may capture functionally meaningful shifts in metal speciation more effectively than total copper or zinc alone [1,7]. Labile copper can participate in redox cycling and metal–protein exchange reactions implicated in cancer metabolism and progression, whereas zinc deficiency compromises Zn-finger transcription factors, antioxidant enzymes, and DNA repair pathways [3,6]. The associations between exCu:Zn and tumor burden observed in this study suggest that this integrated index may capture clinically relevant aspects of systemic metal disequilibrium in CRC.

A novel aspect of this work is the parallel between systemic Cu–Zn imbalance and tissue-level remodeling of epithelial and cytoskeletal proteins associated with epithelial plasticity and EMT-related remodeling [25]. Using paired tumor and matched adjacent mucosa, we observed consistent reductions in vinculin and vimentin in tumor tissue in both sexes, together with a relative increase in E-cadherin, particularly in men. This pattern does not reflect a uniform shift toward a fully mesenchymal phenotype but rather a reorganization of adhesion and cytoskeletal complexes, potentially consistent with partial EMT or hybrid epithelial states described in CRC [12,26,27]. Such states are increasingly recognized as biologically relevant for tumor progression and plasticity. However, because EMT-related proteins were quantified in whole-tissue lysates in the current study, the observed expression patterns may partly reflect differences in stromal and epithelial cellular composition between tumor and adjacent mucosa.

Importantly, systemic Cu–Zn indices—particularly exCu:Zn—were associated with tumor − normal differences in EMT-related proteins. In women, ΔE-cadherin showed strong positive associations with total copper, exCu, and exCu:Zn and inverse associations with zinc, whereas in men ΔE-cadherin was selectively associated with exCu and exCu:Zn. Moreover, exCu:Zn remained positively associated with ΔE-cadherin in women, and similar associations were observed after accounting for age. These findings suggest a potential link between systemic metal disequilibrium and local epithelial remodeling. E-cadherin-based adhesion complexes are sensitive to redox conditions [28] and divalent metal availability [29], and disruption of zinc-dependent structural stability in the presence of excess labile copper may alter junctional organization and epithelial plasticity [30]. While mechanistic causality cannot be inferred from the present data, the concordance between systemic and tissue-level signatures is consistent with a potential relationship between systemic metal disequilibrium and local tissue remodeling that warrants further investigation.

The observed sex-specific patterns further indicate that Cu–Zn imbalance operates within distinct endocrine and metabolic contexts. In women, exCu:Zn was associated with tumor burden and EMT-related remodeling, whereas in men, copper-related indices showed stronger associations with metastatic features. These differences are consistent with known sex-related variation in copper handling, zinc metabolism, and redox regulation [14,31]. The observed associations between exCu-related indices and gonadotropins should be interpreted cautiously and considered exploratory. Rather than supporting specific mechanistic pathways, these findings may reflect broader interactions between hormonal background, systemic metal metabolism, and CRC-related tissue remodeling. Further studies, including direct assessment of endocrine and copper-binding proteins, will be necessary to clarify the biological basis of these sex-specific patterns. In men, the inverse association between LH and zinc and the links between copper-related indices and metastatic status suggest a different balance between redox-active copper pools and zinc-dependent protective mechanisms. These sex-specific patterns may reflect differences in the systemic biochemical environments associated with CRC in women and men, with women having stronger associations with zinc-dependent structural perturbations and men showing stronger associations with copper-driven redox processes.

Strengths of this study include the combined assessment of total copper, exchangeable copper, zinc, and EMT-related proteins in paired tumor − mucosa tissues, together with sex-stratified analyses. A key limitation of this study is the lack of age-matching between CRC patients and healthy controls, as the control group consisted of volunteer blood donors who were significantly younger than the patient cohort. Although all analyses were adjusted for age and stratified by sex, we acknowledge that statistical adjustment may not fully account for differences in age distribution. To address this issue, we performed an additional sensitivity analysis restricting the control group to individuals aged ≥60 years; the results obtained in the age-restricted control subgroup (*n* = 62) were consistent with the findings observed in the full cohort. In addition, although age-adjusted analyses and sensitivity analyses using older controls yielded consistent results, residual confounding cannot be excluded. Factors such as healthy-donor selection bias, age-related physiological changes, systemic inflammation, nutritional status, anemia, and other conditions affecting trace-element homeostasis may have influenced circulating Cu–Zn indices and should be considered in future studies. Nevertheless, these findings should be interpreted with caution, and the case–control comparisons should be considered exploratory rather than definitive. Additional limitations include the cross-sectional design, modest sample size for tissue-based analyses, and the use of matched adjacent mucosa, which may not fully represent truly healthy tissue due to potential field effects. Future studies using age- and clinically matched control groups will be necessary to confirm the diagnostic and biological relevance of these findings. Furthermore, given the exploratory nature of the study and the absence of correction for multiple testing, the reported *p*-values should be interpreted as nominal significance only, and the possibility of false-positive findings cannot be excluded.

## 5. Conclusions

In conclusion, our findings indicate that systemic Cu–Zn disequilibrium, summarized by the serum exCu:Zn ratio, is associated with tumor burden and epithelial remodeling in CRC within a sex-specific context. This integrated metal imbalance may reflect altered competition for thiol-rich ligands and perturbation of zinc-dependent structural complexes in the presence of excess labile copper. Although exploratory, these results provide a rationale for further investigation of exCu:Zn as a candidate biomarker in prospective and risk-stratification settings and for studies aimed at clarifying the biological basis of the observed associations between metal homeostasis, endocrine background, and CRC-related tissue remodeling.

## Figures and Tables

**Table 1:** Group characteristics—CRC vs. controls.

	CRC (*n* = 152)	Control (*n* = 140)	Group Comparisons (*t*-Test)
**Age—mean ± SD**	71.7 ± 12.0	56.0 ± 14.3	t = 10.16, *p* < 0.0001
**Female (%)**	68 (44.7%)	58 (41.4%)	
**Male (%)**	84 (55.3%)	81 (57.9%)	*p* = 0.69 *
**BMI**	25.7 ± 5.41	25.12 ± 6.0	t = 0.86, *p* = 0.39
**Hemoglobin**	11.6 ± 1.9 (*n* = 135)	14.4 ± 1.0	t = −11.26, *p* < 0.0001)
	**Variable**	**Category**	***n***
	**Pathological T stage (pT)**	pT1	10
		pT2	29
		pT3	84
		pT4	29
	**Pathological N stage (pN)**	pN0	82
		pN1	36
		pN2	34
	**Distant metastasis**	M0	135
		M1	17
	**Tumor location**	Right colon	88
		Left colon	48
		Rectum	16

* Sex comparison (chi^2^ test).

**Table 2:** Copper- and zinc-related indices in female CRC patients and controls.

		Variable	Category	*p* Values	Reference Range
**Female**	**Total Cu (µmol/L)**	16.34 (14.30–19.45)	14.00 (12.62–15.75)	0.0061	12.6–24.4 [22]
	**Zn (µmol/L)**	12.00 (9.37–16.40)	17.20 (15.96–18.96)	0.0028	12–24 [22]
	**Cu:Zn ratio**	1.27 (0.98–1.77)	0.84 (0.72–1.02)	0.0008	-
	**exCu (µmol/L)**	1.43 (1.10–1.87)	1.00 (0.80–1.35)	0.1030	0.27–1.90 µmol/L [18]
	**exCu:Zn ratio**	0.12 (0.08–0.15)	0.05 (0.04–0.07)	0.0011	
**Male**		**CRC median** **(Q1–Q3)**	**Control median** **(Q1–Q3)**	***p* (age-adjusted)**	**Normal reference range**
	**Total Cu (µmol/L)**	14.87 (13.08–17.83)	12.78 (11.10–14.48)	<0.0001	11–24 [22]
	**Zn (µmol/L)**	14.40 (11.90–16.72)	19.90 (17.40–21.62)	<0.0001	12–24 [22]
	**Cu:Zn ratio**	1.06 (0.82–1.46)	0.65 (0.54–0.73)	<0.0001	-
	**exCu (µmol/L)**	1.52 (0.90–1.75)	1.00 (0.46–1.41)	<0.0001	0.27–1.90 µmol/L [18]
	**exCu:Zn ratio**	0.10 (0.06–0.14)	0.05 (0.02–0.07)	<0.0001	-

Reference intervals for Cu and Zn were based on clinically validated laboratory standards and published population-based studies [22], whereas the exCu interval was derived from an independent CLSI-based validation study [18].

**Table 3:** Comparison of EMT markers in tumor and matched adjacent non-neoplastic mucosa.

Marker	Sex	Adjacent Non-Neoplastic Mucosa Median (Q1–Q3)	Tumor Median (Q1–Q3)	Within-Patient Differential (Δ = Tumor − Adjacent) Median (Q1–Q3)	*p* (Wilcoxon)
**MEMO1**	Females	0.85 (0.62–1.07)	1.05 (0.83–1.25)	0.08 (−0.09–0.42)	0.115
	Males	0.97 (0.68–1.37)	1.00 (0.77–1.19)	0.00 (−0.29–0.30)	0.588
**Vinculin**	Females	1.18 (0.96–1.41)	0.64 (0.42–1.07)	−0.52 (−0.81–−0.16)	0.000
	Males	1.25 (1.02–1.89)	0.70 (0.46–0.92)	−0.62 (−0.94–−0.13)	<0.0001
**Fibronectin**	Females	1.23 (0.38–1.79)	1.15 (0.78–1.49)	0.10 (−0.65–0.81)	0.663
	Males	0.76 (0.35–1.26)	0.74 (0.48–1.62)	0.08 (−0.38–0.62)	0.271
**E-cadherin**	Females	0.94 (0.66–1.18)	1.13 (0.63–1.51)	0.14 (−0.29–0.57)	0.201
	Males	0.67 (0.35–1.11)	1.06 (0.70–1.53)	0.33 (−0.13–0.87)	0.002
**Vimentin**	Females	1.66 (0.76–3.05)	0.31 (0.22–0.76)	−1.25 (−3.05–−0.34)	0.006
	Males	2.13 (1.33–4.76)	0.48 (0.20–1.09)	−1.54 (−4.79–−0.42)	<0.0001

Values are expressed as median (25th–75th percentile). Tumor vs. matched adjacent non-neoplastic mucosa were compared using the Wilcoxon signed-rank test, calculated separately in women and men. A two-sided *p* value < 0.05 was considered statistically significant. Tissue-based analyses were performed on the subset of patients with complete paired tissue, serum, hemoglobin, and hormone profile data. Effective sample sizes for each marker are reported in Supplementary Table S2.

## Data Availability

Data supporting the reported results can be provided by contacting the corresponding authors. All study data will be deposited in a public repository upon the conclusion of the study as soon as the full results are finalized.

## Supplementary Materials

The following supporting information can be downloaded at https://www.mdpi.com/article/10.3390/biom16060878/s1, Supplementary Table S1. (a) Serum hormones and CEA in female CRC patients and controls; (b) Serum hormones and CEA in male CRC patients and controls. Supplementary Table S2. Effective sample sizes for tissue-based analyses. Supplementary Figure S1. Spearman correlations between tumor clinical indices and copper/zinc-related variables, stratified by sex. Heatmaps display Spearman correlation coefficients (ρ) between tumor clinical indices (columns: Tumor size, pT stage, pN stage, Metastasis, TNM stage) and copper/zinc-related variables (rows: Total Cu, Zn, Cu:Zn ratio, exCu, exCu:Zn ratio, Tissue Cu [Δ tumor − normal]) in (A) females and (B) males with colon cancer. Colors indicate the magnitude and direction of Spearman ρ (blue = negative, red = positive), as shown by the color scale. Numerical values inside each tile correspond to the correlation coefficients; tiles marked with an asterisk (*) denote statistically significant correlations (*p* < 0.05). Supplementary Figure S2. Age-adjusted associations between copper/zinc indices and circulating hormones, stratified by sex. Heatmaps show age-adjusted Spearman correlation coefficients (ρ) between serum/tissue copper–zinc indices (rows) and circulating hormones (columns) in (A) females and (B) males. Copper/zinc-related variables include total serum copper (Total Cu), serum zinc (Zn), the serum Cu:Zn ratio (Cu:Zn ratio), exchangeable copper (exCu), the exchangeable copper-to-zinc ratio (exCu:Zn ratio), and the tumor–normal difference in tissue copper content (Tissue Cu [Δ tumor–normal]). Hormones include FSH, estradiol, LH, progesterone, and testosterone, while CEA was assessed as a clinical tumor marker. All variables were first residualized for age by linear regression, and Spearman correlations were computed on the residuals. Colors represent ρ values (blue = negative, red = positive) as indicated by the color scale; numerical values inside each tile indicate the corresponding correlation coefficient, and tiles marked with an asterisk (*) denote statistically significant correlations (*p* < 0.05). Supplementary Material S2: The original Western Blot images.
