# Supplementary material for: The Exchangeable Copper–Zinc Ratio Links Sex Hormones, Tumor Burden, and Epithelial Remodeling in Colorectal Cancer
Source: Biomolecules. 2026 Jun 15;16(6):878. doi: 10.3390/biom16060878 (PMC13297341; doi:10.3390/biom16060878)

E-cadherin Male

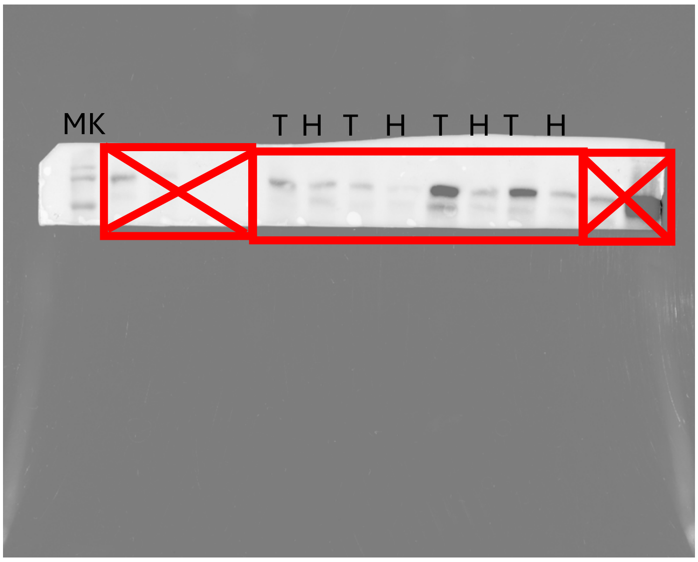

E-cadherin Female

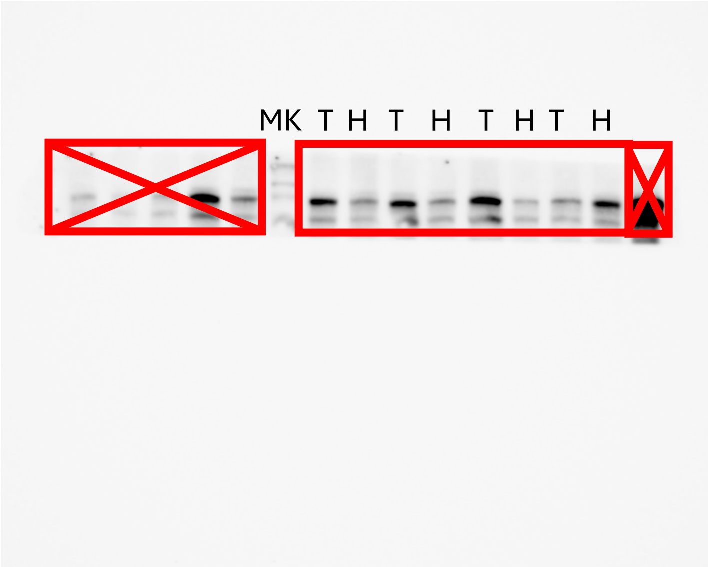

Fibronectin Male

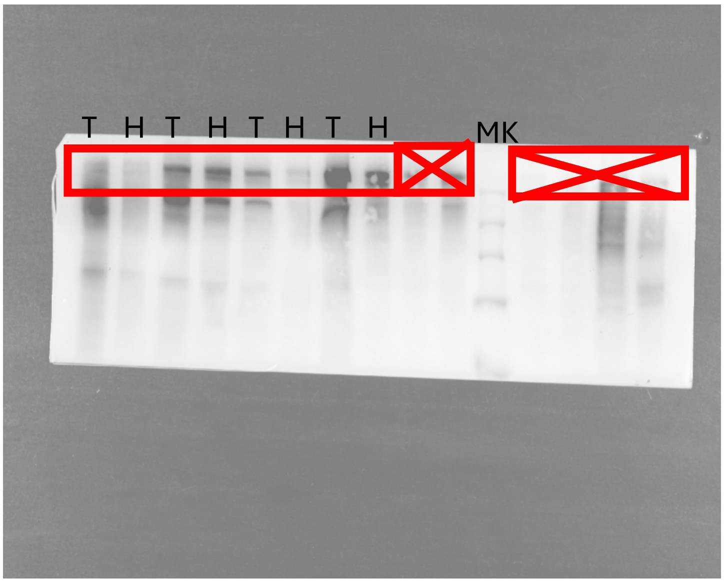

Fibronectin Female

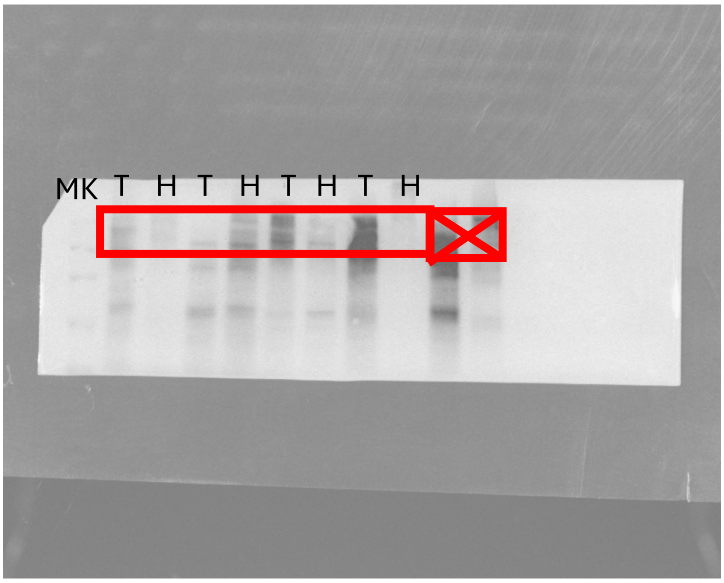

Vimentin male

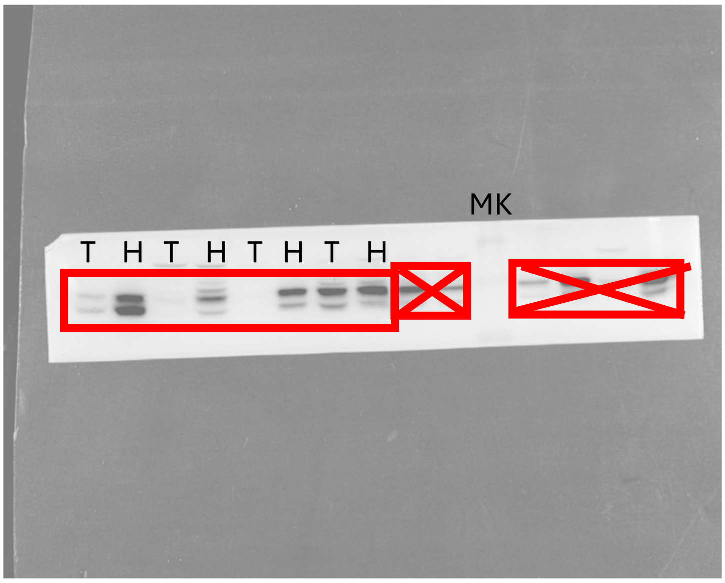

Vimentin Female

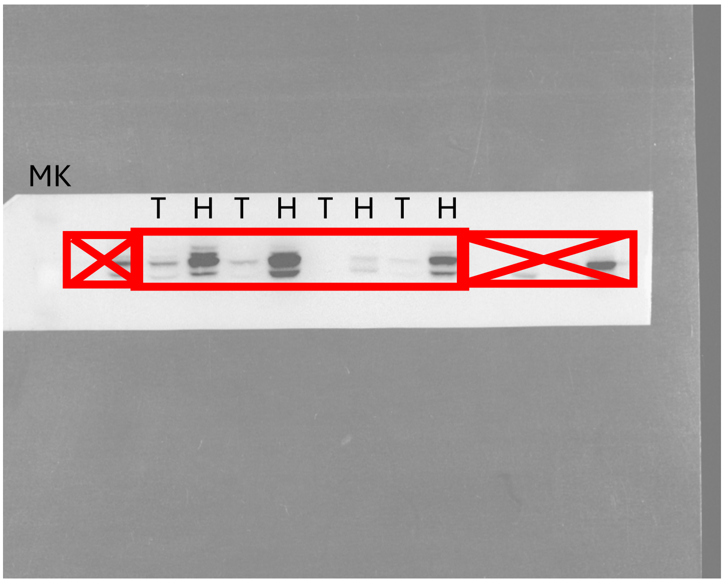

Vinculin Male

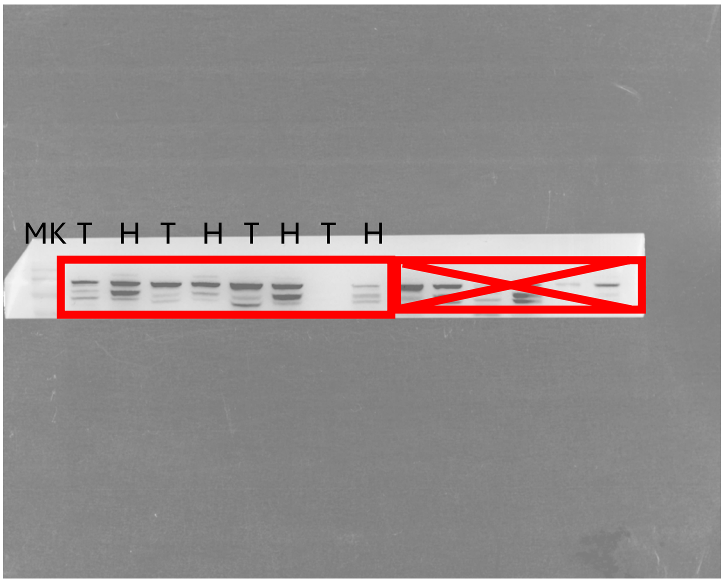

Vinculin Female

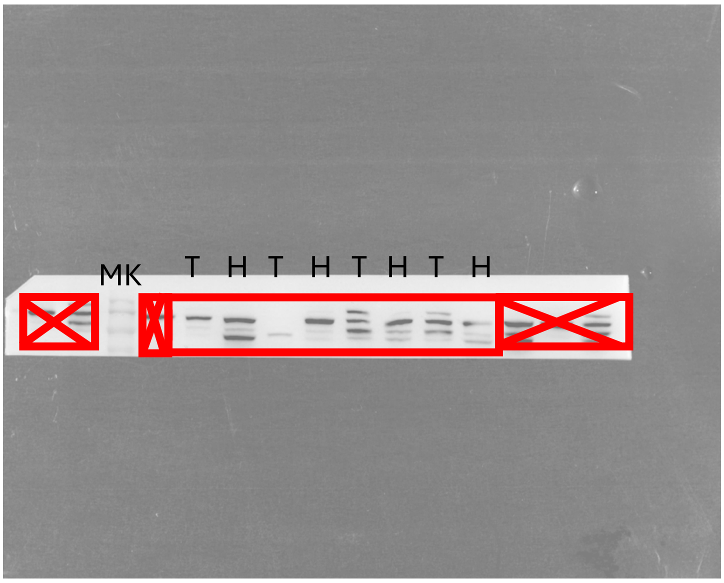

MEMO1 Male

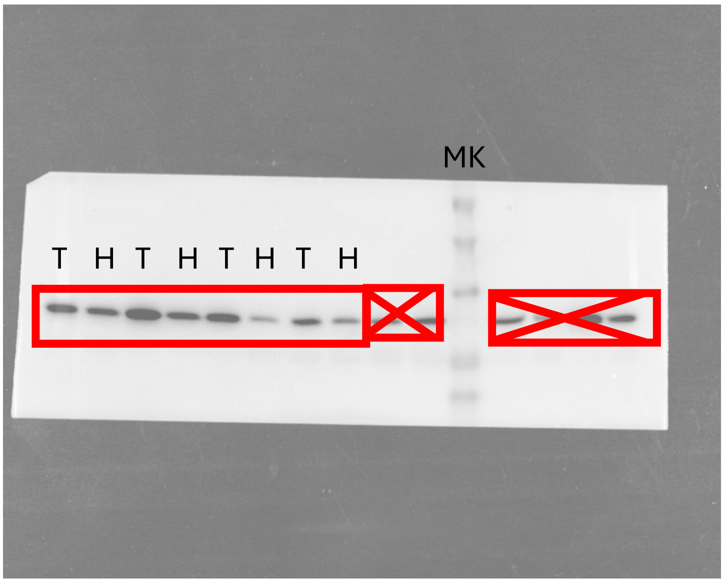

MEMO1 Female

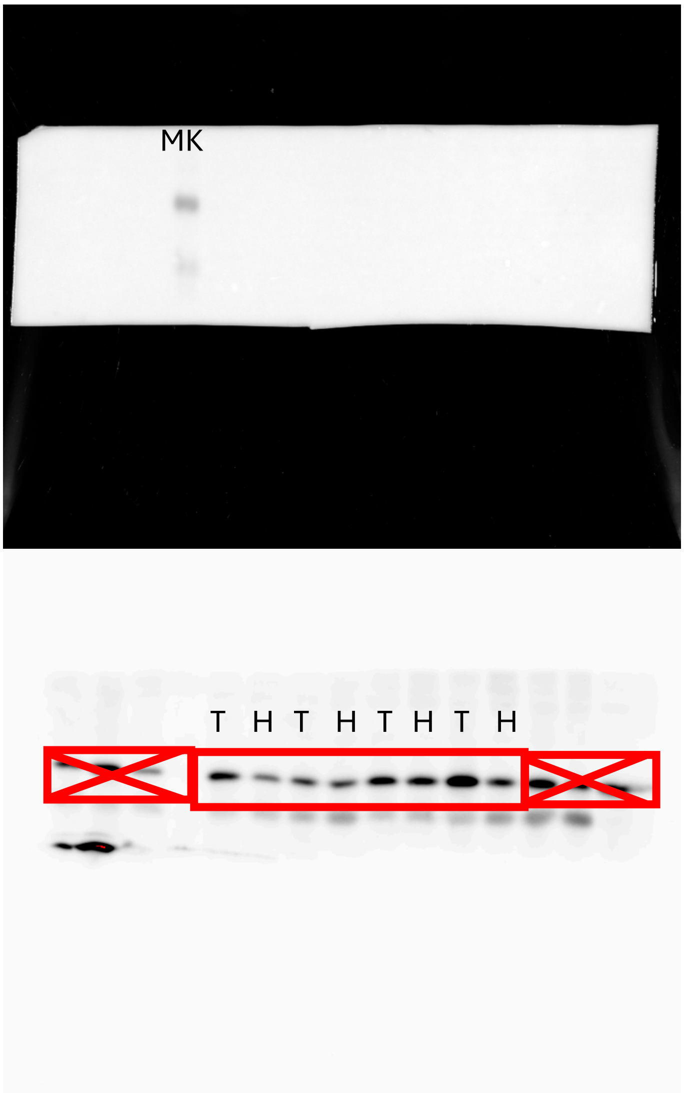

Actin Male

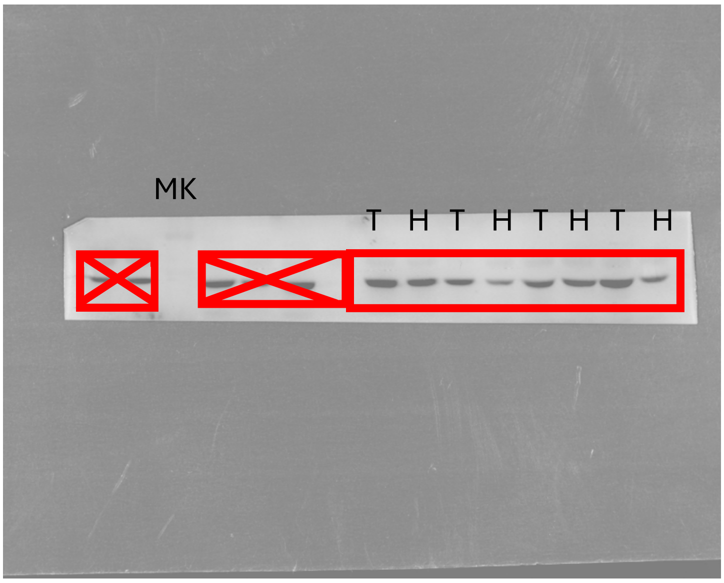

Actin Female

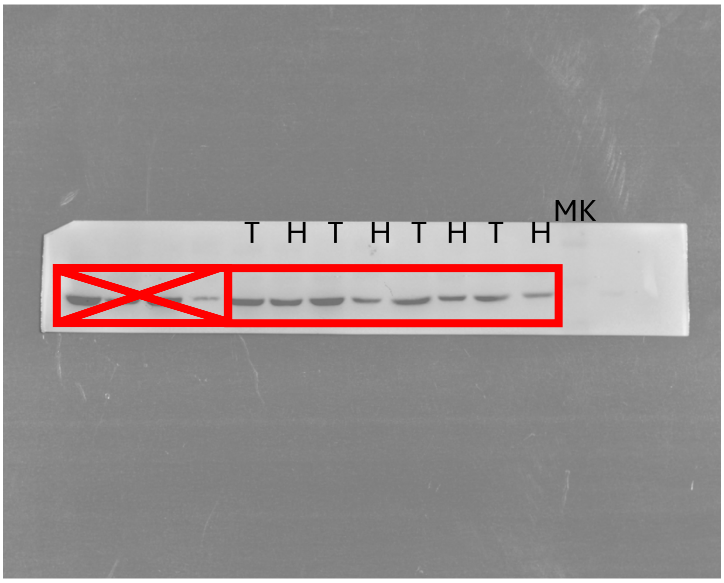

Supplement: Supplementary file 1 [file biomolecules-16-00878-s001.zip › biomolecules-4261791-Supplementary Material S2-The original Western Blot images.pdf]
