# Supplementary material for: The Exchangeable Copper–Zinc Ratio Links Sex Hormones, Tumor Burden, and Epithelial Remodeling in Colorectal Cancer
Source: Biomolecules. 2026 Jun 15;16(6):878. doi: 10.3390/biom16060878 (PMC13297341; doi:10.3390/biom16060878)
Supplement: Supplementary file 1 [file biomolecules-16-00878-s001.zip › biomolecules-4261791-supplementary.pdf]

Supplementary Materials of the Study: The Exchangeable Copper–Zinc Ratio Links Sex Hormones, Tumor Burden, and Epithelial Remodeling in Colorectal Cancer

Supplementary Table S1-a. Serum hormones and CEA in female CRC patients and controls

	CRC median (Q1–Q3)	Control median (Q1–Q3)	p (age-adjusted)*	Normal reference range
**FSH (IU/L)**	47.93 (30.30–68.59)	71.84 (52.42–83.71)	0.0052	19.3–100.6
**Estradiol (pg/mL)**	17.27 (13.00–22.75)	10.00 (10.00–14.43)	0.0227	0–39.5
**LH (IU/L)**	17.13 (10.54–20.88)	21.30 (16.58–26.30)	0.0373	14.2–52.3
**Progesterone (ng/mL)**	0.10 (0.06–0.45)	0.10 (0.06–0.10)	0.0015	0.15–0.8
**Testosterone (nmol/L)**	0.86 (0.63–1.23)	0.99 (0.80–1.20)	0.4012	0.52-2.43
**CEA (ng/mL)**	2.55 (1.73–5.59)	1.94 (1.73–2.97)	0.1152	0-2.9

Supplementary Table S1-b. Serum hormones and CEA in male CRC patients and controls

	CRC median (Q1–Q3)	Control median (Q1–Q3)	p (age-adjusted)*	Normal reference range
**FSH (IU/L)**	7.50 (4.19–14.29)	4.45 (3.34–5.88)	0.5773	1.5–12.4
**Estradiol (pg/mL)**	28.00 (21.53–34.53)	30.00 (23.90–33.53)	0.1078	0–39.5
**LH (IU/L)**	4.25 (3.52–7.13)	3.40 (3.00–3.95)	0.1386	1.8–8.6
**Progesterone (ng/mL)**	0.17 (0.10–0.20)	0.20 (0.17–0.20)	0.7656	0.15–0.8
**Testosterone (nmol/L)**	13.64 (8.83–17.89)	17.43 (14.21–20.28)	0.2202	8.6-29.0
**CEA (ng/mL)**	2.83 (1.73–6.62)	1.73 (1.73–2.68)	0.0909	0-2.9

Values are expressed as median (25th–75th percentile). *p-values from linear regression models with $\log_{10}$-transformed hormone levels as the dependent variable, diagnosis (CRC vs control) as fixed factor, and age as covariate, fitted separately in females and males.

Supplementary Table S2.

Effective sample sizes for tissue-based analyses.

Marker	Total n	Females	Males
MEMO1	78	24	54
Vinculin	78	23	55
Fibronectin	79	24	55
E-cadherin	77	24	53
Vimentin	64	22	47

Tissue-based analyses were performed on patients with complete paired tissue, serum, hemoglobin, and hormone profile data.

Supplementary Figure S1

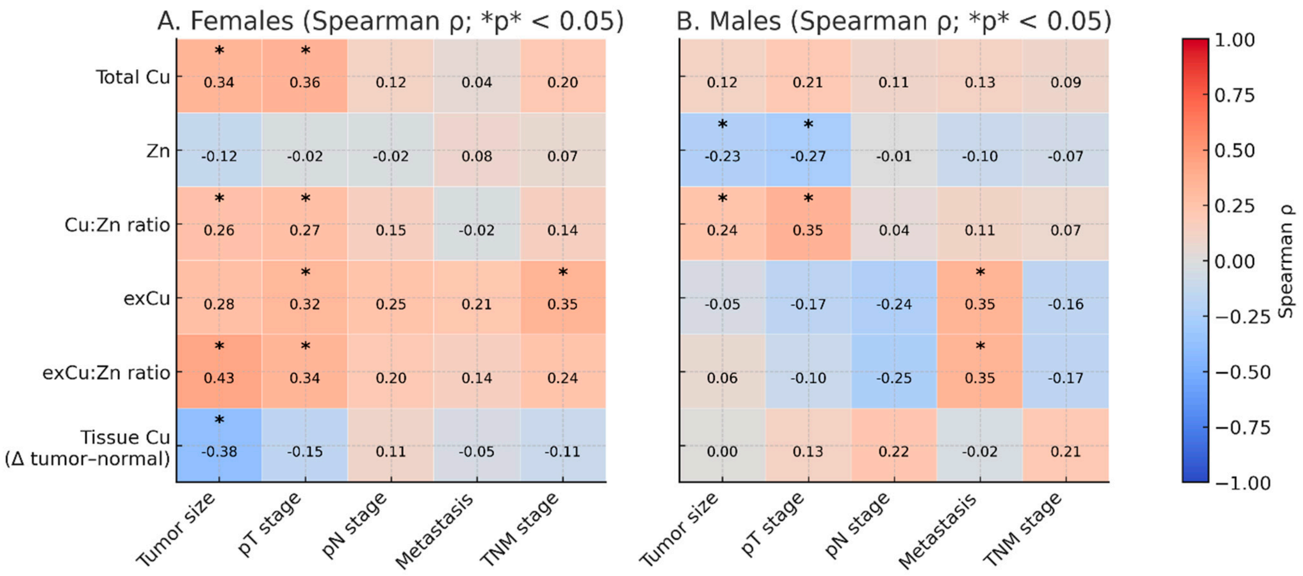

Supplementary Figure S1. *Spearman correlations between tumor clinical indices and copper/zinc-related variables, stratified by sex.* Heatmaps display Spearman correlation coefficients (ρ) between tumor clinical indices (columns: Tumor size, pT stage, pN stage, Metastasis, TNM stage) and copper/zinc-related variables (rows: Total Cu, Zn, Cu:Zn ratio, exCu, exCu:Zn ratio, Tissue Cu [Δ tumor–normal]) in (A) females and (B) males with colon cancer. Colors indicate the magnitude and direction of Spearman ρ (blue = negative, red = positive), as shown by the color scale. Numerical values inside each tile correspond to the correlation coefficients; tiles marked with an asterisk (*) denote statistically significant correlations ($p < 0.05$).

Supplementary Figure S2

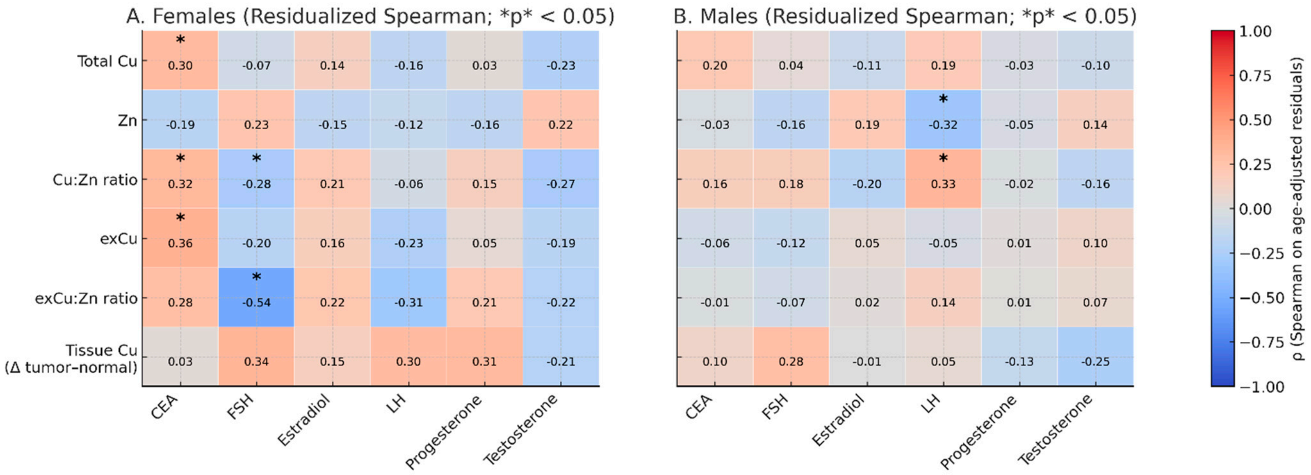

Supplementary Figure S2. *Age-adjusted associations between copper/zinc indices and circulating hormones, stratified by sex.* Heatmaps show age-adjusted Spearman correlation coefficients (ρ) between serum/tissue copper–zinc indices (rows) and circulating hormones (columns) in (A) females and (B) males. Copper/zinc-related variables include total serum copper (Total Cu), serum zinc (Zn), the serum Cu:Zn ratio (Cu:Zn ratio), exchangeable copper (exCu), the exchangeable copper-to-zinc ratio (exCu:Zn ratio), and the tumor–normal difference in tissue copper content (Tissue Cu [Δ tumor–normal]). Hormones include FSH, estradiol, LH, progesterone, and testosterone, while CEA was assessed as a clinical tumor marker. All variables were first residualized for age by linear regression, and Spearman correlations were computed on the residuals. Colors represent ρ values (blue = negative, red = positive) as indicated by the color scale; numerical values inside each tile indicate the corresponding correlation coefficient, and tiles marked with an asterisk (*) denote statistically significant correlations ($p < 0.05$).
